# Does Kinesio Taping Enhance Exercise Performance and Recovery in Healthy Males Under Heat Stress?

**DOI:** 10.3390/jcm14082631

**Published:** 2025-04-11

**Authors:** Yongsuk Seo, Yunbin Lee, Somang Son, Jooyoung Lee, Jihwan Park, Daetaek Lee, Se-Young Seon, Somi Yun

**Affiliations:** Exercise Physiology Laboratory, Kookmin University, Seoul 02707, Republic of Korea; yseokss@kookmin.ac.kr (Y.S.); aycyb@kookmin.ac.kr (Y.L.); somang5014@kookmin.ac.kr (S.S.); bosukjy@naver.com (J.L.); parkjh9992@kookmin.ac.kr (J.P.); dtlee@kookmin.ac.kr (D.L.)

**Keywords:** blood flow, exercise performance, heat, Kinesio taping, recovery

## Abstract

**Backgrounds/Objectives:** The purpose of this study was to compare blood flow and exercise performance in high-temperature environments with Kinesio taping. **Methods:** Ten heathy men performed a Bruce treadmill test until volitional fatigue in a heated environment (35 °C, 50% relative humidity), with (KTC) and without Kinesio taping (UTC). Blood flow (BF), maximal oxygen uptake (VO_2_max), heart rate (HR), and blood lactate (BL) were measured at baseline, after 30 min of acclimation, during treadmill exercise, and after 30 min of recovery. **Results:** Baseline and resting measurements of BF, VO_2_max, HR, and BL were similar between conditions (*p* > 0.05). The results revealed no significant differences in any variables between conditions (*p* > 0.05), except for BF. BF was significantly higher in the KTC compared to the UTC during exercise (*p* < 0.05). VO_2_, HR, and BL significantly increased during exercise, immediately post-exercise, 10 min post-exercise, and 30 min post-exercise compared to baseline in both KTC and UTC conditions (all *p* < 0.05). When Kinesio tape was applied, BF significantly increased during and after exercise. However, VO_2_, HR, and BL did not differ between conditions. **Conclusions:** These results showed no evidence that Kinesio tape improves exercise performance or recovery in heat conditions.

## 1. Introduction

Kinesio taping (KT) is recognized as a versatile method widely used in sports to enhance recovery, prevent injuries, and improve exercise performance. Initially developed for therapeutic purposes, KT has been applied for functions such as pain relief, muscle stabilization, and injury prevention. Research has highlighted KT’s potential to improve local blood flow (BF) and thermoregulation efficiency [1,2].

The proposed mechanism of KT involves creating convolutions on stretched soft tissues, lifting the skin, and enhancing underlying BF and lymphatic circulation [3,4]. Craighead et al. (2017) demonstrated increased local BF after KT application and others reported enhanced muscle activation [5,6,7]. These findings suggest that KT may optimize circulation, reduce metabolic strain, and potentially improve exercise performance under heat stress. However, the evidence remains inconclusive. A review by Reneker et al. (2018) concluded that there is no compelling evidence to support KT consistently enhancing exercise performance, noting that studies claiming improvements often lacked proper blinding and failed to account for placebo effects [8]. Previous systematic reviews have identified significant methodological limitations within the existing kinesiology taping (KT) literature. Williams et al. (2012) reported that inadequate blinding procedures, small sample sizes, and the potential influence of placebo effects undermine the internal validity of many KT studies [9]. Parreira et al. (2014) similarly noted inconsistencies in study design and insufficient clinical evidence to support the efficacy of KT [10]. Furthermore, Montalvo et al. (2014) reported that although some studies reported positive outcomes, the lack of methodological consistency raises concerns regarding the reliability of these findings [11]. These conflicting findings underscore the need for further investigation into KT’s effects.

While many KT studies have examined its effects under general conditions, limited research has explored its efficacy in high-temperature environments. Liu et al. (2020) found that KT improved skin temperature and blood circulation [12], but these findings were obtained under standard conditions rather than during heat stress. Exercising in hot environments disrupts thermoregulation and substrate metabolism, exacerbating physiological strain. Heat stress elevates body temperature and induces dehydration, reducing plasma volume, cardiac output, and oxygen delivery, which collectively impair exercise performance [13,14]. In addition, hyperthermia accelerates carbohydrate metabolism, increasing intramuscular glycogen utilization and blood lactate (BL) accumulation, thereby contributing to early fatigue [15]. In this context, KT has been proposed to facilitate blood flow (BF) and support muscle function, potentially counteracting some physiological impairments induced by heat stress.

By promoting localized circulation and possibly enhancing neuromuscular activation, KT may aid in thermoregulatory efficiency and delay fatigue during exercise in high-temperature conditions. However, these effects remain speculative, and evidence under thermally stressful conditions is limited. A previous study reported that KT application resulted in a significant increase in waist skin temperature after 10 min, suggesting a potential enhancement in local microcirculation [12]. In contrast, other studies have shown that KT does not significantly influence cutaneous blood microcirculation in healthy individuals at rest, nor does it lead to measurable changes in blood flow, muscle volume, or endurance in the gastrocnemius muscle [16,17].

Given these inconsistent findings and the limited research conducted under heat stress conditions, determining whether KT can enhance BF, maintain thermoregulation, and mitigate metabolic strain during exercise in high-temperature environments remains an important area for investigation.

Such findings would be particularly relevant not only for athletes but also for occupational groups exposed to extreme heat. This study aims to evaluate the effects of KT on BF and exercise performance in high-temperature environments. By examining the physiological mechanisms underlying KT’s impact under heat stress, the current research seeks to provide foundational data to optimize human efficiency in both sports and industrial settings through practical applications. While KT has been proposed to offer physiological benefits, it is hypothesized that KT application will not significantly improve blood flow, enhance exercise performance, or aid recovery during exercise in high-temperature environments.

## 2. Methods

### 2.1. Participants

Participant characteristics are shown in Table 1. Ten healthy, physically active, non-smoking men volunteered for this study. All participants completed a health history questionnaire prior to participation and were excluded if they reported any cardiovascular or musculoskeletal disorders, or recent injuries within the past three months. While all participants reported engaging in moderate-intensity physical activity at least twice per week, none had a history of structured athletic training or participation in competitive sports.

Participants were also excluded if they had been acclimatized to heat within the three months prior to the study. Before participating in the study, all participants were provided with the purpose, procedures, potential risks, and benefits of the study. After confirming that they fully understood the information provided, each participant provided written and verbal informed consent. All participants were instructed to refrain from strenuous exercise and alcohol consumption for at least 24 h before each experimental trial. During the prescreening session, participants’ height and weight were measured and recorded using a stadiometer (DS-102, JENIX, Seoul, Republic of Korea) and a scale (DB-150, CAS, Yangju-si, Gyeonggi-do, Republic of Korea), respectively. Body composition was assessed using a bioelectrical impedance analyzer (InBody 570, InBody, Seoul, Republic of Korea) to measure weight (kg), muscle mass (kg), fat-free mass (kg), and body fat mass (kg).

This research adhered to the principles outlined in the Helsinki Declaration and the protocol was approved by the Institutional Review Board (IRB) of Kookmin University Research Ethics Committee (KMU-202406-HR-418; Date of approval: 21 June 2024).

### 2.2. Experimental Design

This study employed a repeated measures, within-subject design with a counterbalanced order across participants to minimize potential order effects. The protocol included two experimental trials: one with KT and one without KT in the heat. Environmental conditions were set to a temperature of 35 °C and a relative humidity of 50%. An electronic thermo-hygrometer (SK-L200TH, SATO KEIRYOKI, Tokyo, Japan) recorded environmental temperature data in real time, with measurements taken in seconds from the start to the end of the experiment. Each trial was separated by a minimum of 7 days to ensure full recovery. During both conditions, participants performed on an electronic treadmill using a progressive overload method based on the Bruce Protocol. They began at stage 1 (speed: 2.7 km/h, inclined: 10%) for 3 min. Every 3 min, both the incline and speed were progressively increased. The test was terminated when the participant reached volitional fatigue and could no longer continue.

### 2.3. Experimental Procedure

All experimental procedures were conducted in the exercise physiology laboratory at Kookmin University. On the days of the two experimental trials, participants arrived at the lab at the same time of day for consistency.

Upon arrival, participants wore T-shirts, shorts, and self-selected athletic shoes. They were then equipped with a heart rate (HR) monitor and a laser Doppler probe. Each participant was positioned in a supine position with the hip flexed at 30° and the knee flexed at 60°.

**Figure 1:**
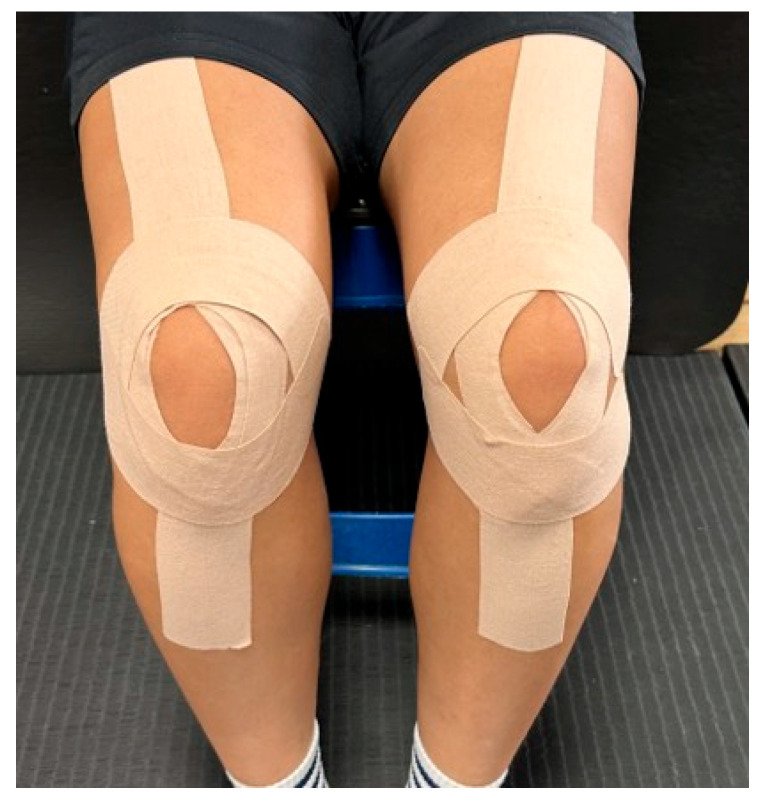
The application of Kinesio tape.

The KT (MKLA-10701, Naum Care, Seongnam, Republic of Korea) was applied 10 cm below the anterior superior iliac spine, with the first 5 cm applied without tension (Figure 1). The tape was then stretched to 120% and applied in a straight line from the hip joint to the patella, secured above and below the patella. After application, the tape’s adhesion was checked, and it was ensured that no discomfort was present on the skin. This technique was chosen based on previous studies indicating its effects of reducing pain [18,19], enhancing muscle strength [20], and improving blood flow [5]. Due to the visible and tactile nature of KT application, participant blinding was not feasible.

Measurements of BL, BF, and core temperature were taken before exercise, immediately after exercise, at 10 min recovery, and at 30 min recovery.

### 2.4. Measurements and Instrumentation

Oxygen uptake was continuously monitored using a portable wireless gas analyzer (K5, Cosmed, Rome, Italy). The O_2_ sensor was connected to a flowmeter and a mask, allowing for the collection of data on the concentration of inhaled oxygen and exhaled carbon dioxide. VO_2_ is defined as the amount of oxygen an individual can utilize at a given exercise intensity over time. VO_2_max is further defined as the highest value recorded during exercise, confirmed by reaching a respiratory exchange ratio (RER) greater than 1.1, a rating of perceived exertion (RPE) above 17, or a plateau in heart rate or VO_2_. HR was monitored using a Polar band-type HR sensor (H10, Polar, Kempele, Finland), with real-time data transmission to a receiver (Polar Vantage M2, Polar, Kempele, Finland).

BL levels were measured from finger-prick capillary blood samples using the Accutrend Plus analyzer (Roche, Basel, Switzerland). Samples were taken at predetermined time points, with two samples averaged at each point. The lactate analyzer was calibrated with standard solutions before and after each test. To assess changes in BL concentration at the 15 min and 30 min recovery marks, the percentage decrease in mean BL concentration was calculated using following formular.

$$\mathrm{Blood}\mathrm{lactate}\mathrm{clearance}\mathrm{rate}\left(\%\right)=\frac{All\;out-Recovery}{All\;out-Rest}\times 100$$

BF was assessed using a laser Doppler flow probe (ALF21R, ADVANCE, Tokyo, Japan). The input voltage was adjusted to 100 V, and the device was automatically calibrated to ensure that the displayed value matched the manufacturer’s predefined standard (Flow: 10.0 mL/min/100 g). After confirming calibration, the probe was then positioned on the skin over the rectus femoris muscle. The laser light was scattered and reflected by red blood cells within the skin and blood vessels. The BF analyzer calculated the BF by multiplying the estimated cross-sectional area of the vessel by the measured BF velocity.

Core temperature was measured using an ear thermometer (IRT-4520, Braun GMBH, Kronberg, Germany) placed in the ear canal. The participant’s ear was gently pulled upward or backward to straighten the ear canal, and two readings were taken. The average of these readings was recorded.

### 2.5. Data Analysis

Using a statistical software package (SPSS 29.0), two-way repeated measures ANOVA was utilized to compare physiological measurements between taping and untapping conditions. When the ANOVA indicated a significant main effect, post hoc pairwise comparisons with the least significant difference (LSD) were carried out to identify the difference between conditions.

Exploratory bivariate correlations were conducted to determine the relationship between BF and VO_2_max, duration, HR, and BL. All data are presented as mean ± standard deviation (SD) and the level of significance was set at α ≤ 0.05 for all statistical comparisons.

## 3. Results

The baseline and resting measurements for BF, VO_2_max, HR, core temperature, and BL were similar between the KTC and UTC conditions, indicating equal variance. Environmental conditions for both conditions are displayed in Table 2.

### 3.1. Blood Flow

**Figure 2:**
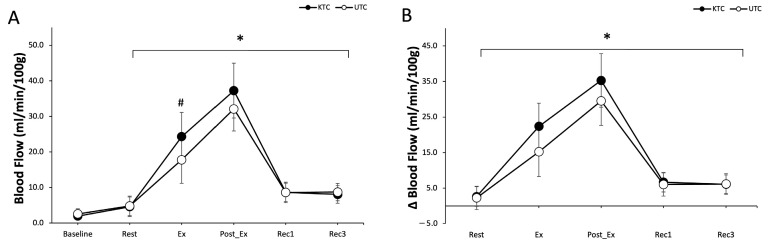
(**A**) Blood flow (ml/min/100 g) at baseline, rest, exercise (Ex), post-exercise (Post-Ex), Recovery 1 (Rec1), and Recovery 3 (Rec3) for KTC (filled circles) and UTC (open circles). (**B**) Changes in blood flow (Δ Blood Flow, mL/min/100 g) at rest, exercise (Ex), post-exercise (Post-Ex), Recovery 1 (Rec1), and Recovery 3 (Rec3) for KTC (filled circles) and UTC (open circles). KTC—Kinesio tape condition; UTC—untreated condition. Values are mean ± SD. * *p* ≤ 0.05 vs. baseline; # *p* ≤ 0.05 vs. UTC.

BF showed a significant main effect for conditions (F = 7.858, *p* = 0.021) and time (F = 79.343, *p* = 0.001). However, no significant condition-by-time interaction was observed (F = 1.937, *p* = 0.243) (Figure 2A). Post hoc analyses revealed significantly higher BF during exercise (24.3 ± 6.8 vs. 17.8 ± 6.6 mL/min/100 g, Cohen’s d = −0.83) and immediately post-exercise (37.2 ± 7.7 vs. 32.1 ± 6.2 mL/min/100 g; Cohen’s d = 0.48), as well as at 10 min post-exercise (8.6 ± 2.6 vs. 8.6 ± 2.9 mL/min/100 g; Cohen’s d = −0.01) and 30 min post-exercise (8.1 ± 2.5 vs. 8.7 ± 2.4 mL/min/100 g; Cohen’s d = 0.18) compared to baseline for the KTC and UTC conditions, respectively (all *p* < 0.05). Furthermore, BF was significantly higher in KTC compared to UTC during exercise (*p* < 0.05) (Figure 2A). 

Similarly, the change in BF from baseline showed a significant main effect for both conditions (F = 6.506, *p* = 0.031) and time (F = 59.685, *p* = 0.001). However, there was no significant condition-by-time interaction (F = 2.142, *p* = 0.193) (Figure 2B). Post hoc analyses revealed significantly greater changes in BF during exercise (22.4 ± 6.5 vs. 15.2 ± 7.0 mL/min/100 g), immediately post-exercise (35.3 ± 7.5 vs. 29.5 ± 6.9 mL/min/100 g), at 10 min post-exercise (6.7 ± 2.7 vs. 6.0 ± 3.3 mL/min/100 g), and at 30 min post-exercise (6.1 ± 2.6 vs. 6.1 ± 2.9 mL/min/100 g) compared to rest (all *p* < 0.05) (Figure 2B).

### 3.2. Exercise Time, VO_2_max, and Heart Rate

**Figure 3:**
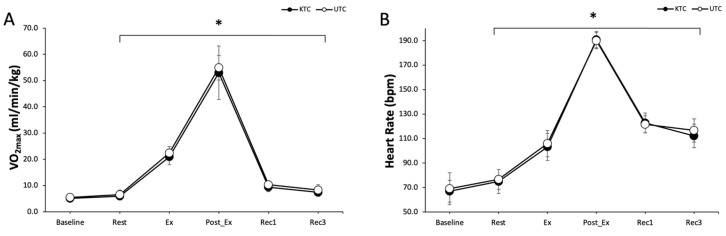
(**A**) Oxygen uptake at baseline, rest, exercise (Ex), post-exercise (Post-Ex), Recovery 1 (Rec1), and Recovery 3 (Rec3) for KTC (filled circles) and UTC (open circles). (**B**) Heart rate at rest, exercise (Ex), post-exercise (Post-Ex), Recovery 1 (Rec1), and Recovery 3 (Rec3) for KTC (filled circles) and UTC (open circles). KTC—Kinesio tape condition; UTC—untreated condition. Values are mean ± SD. * *p* ≤ 0.05 vs. baseline.

Total exercise time did not differ between conditions (UTC: 766.7 ± 46.0 vs. KTC: 775.4 ± 45.5 s) (t = −0.801, *p* = 0.444). VO_2_max also did not differ between conditions (F = 2.169, *p* = 0.175). However, there was a significant main effect for time (F = 189.029, *p* = 0.001). No significant condition-by-time interaction was found (F = 0.395, *p* = 0.834) (Figure 3A).

Post hoc analyses revealed that VO_2_max significantly increased during exercise (20.9 ± 3.0 vs. 22.4 ± 2.5 mL/min/kg), immediately post-exercise (53.0 ± 10.2 vs. 54.9 ± 4.7 mL/min/kg), 10 min post-exercise (9.4 ± 1.7 vs. 10.3 ± 1.2 mL/min/kg), and 30 min post-exercise (7.4 ± 1.5 vs. 8.3 ± 2.0 mL/min/kg) compared to baseline in the KTC and UTC conditions, respectively (all *p* < 0.05).

HR showed no significant main effect for condition (F = 0.653, *p* = 0.440) or time (F = 626.707, *p* = 0.001). There was no significant condition-by-time interaction (F = 1.116, *p* = 0.366) (Figure 3B). Post hoc analyses revealed that HR significantly increased during exercise (103.2 ± 11.1 vs. 106.0 ± 10.8 bpm), immediately post-exercise (190.8 ± 6.8 vs. 190.0 ± 6.6 bpm), 10 min post-exercise (122.8 ± 8.0 vs. 121.6 ± 6.9 bpm), and 30 min post-exercise (112.2 ± 9.6 vs. 116.8 ± 9.4 bpm) compared to baseline in the KTC and UTC conditions, respectively (all *p* < 0.05).

### 3.3. Blood Lactate and Lactate Clearance Rate

**Figure 4:**
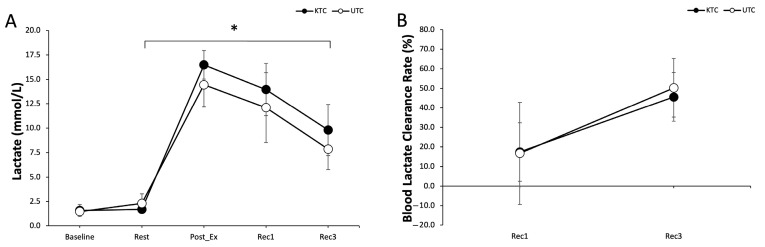
(**A**) Blood lactate concentration (mmol/L) at baseline, rest, post-exercise (Post-Ex), Recovery 1 (Rec1), and Recovery 3 (Rec3) for KTC (filled circles) and UTC (open circles). (**B**) Blood lactate clearance rate (%) at Recovery 1 (Rec1) and Recovery 3 (Rec3) for KTC (filled circles) and UTC (open circles). KTC—Kinesio tape condition; UTC—untreated condition. Values are mean ± SD. * *p* ≤ 0.05 vs. baseline.

BL levels showed no significant main effect for conditions (F = 3.091, *p* = 0.113) but there was a significant main effect for time (F = 638.578, *p* = 0.001). No significant condition-by-time interaction was observed (F = 1.024, *p* = 0.466) (Figure 4A). Post hoc analyses revealed that BL significantly increased immediately post-exercise (16.5 ± 1.5 and 14.5 ± 2.3 mmol/L for KTC and UTC, respectively), 10 min post-exercise (14.0 ± 2.7 and 12.1 ± 3.6 mmol/L), and 30 min post-exercise (9.8 ± 2.6 and 7.9 ± 2.1 mmol/L) compared with baseline values in both KTC and UTC (all *p* < 0.05) (Figure 4A).

The rate of recovery in BL showed a main effect for time (F = 289.184, *p* ≤ 0.001) but no significant main effect for conditions (F = 2.207, *p* = 0.172). There was also no significant condition-by-time interaction (F = 1.809, *p* = 0.169). Post hoc analyses revealed a significant difference between Rec1 and Rec3 in both conditions (*p* < 0.001), although no significant differences were found between conditions at any recovery time point (Figure 4B).

### 3.4. Perceived Exertion, Thermal Sensation, and Eardrum Temperature

The rating of perceived exertion showed no significant main effect for conditions (F = 0.205, *p* = 0.661), but there was a significant main effect for time (F = 76.310, *p* = 0.001). No significant condition-by-time interaction was observed (F = 0.530, *p* = 0.752) (Table 3).

Thermal sensation revealed no significant main effect for conditions (F = 0.584, *p* = 0.464) but there was a significant main effect for time (F = 55.671, *p* = 0.001). There was no significant condition-by-time interaction (F = 2.240, *p* = 0.067) (Table 3).

Eardrum temperature indicated no significant main effect for the conditions (F = 0.078, *p* = 0.786) and a significant main effect for time (F = 44.920, *p* = 0.001). No significant condition-by-time interaction was found (F = 0.348, *p* = 0.881) (Table 3).

In the KTC condition, BF showed a significant negative correlation with the duration of exercise (r = −0.1) and significant positive correlations with VO_2_max (r = 0.883), HR (r = 0.774), and lactate (r = 0.749), all with *p* < 0.01. In the UTC condition, BF exhibited significant positive correlations with VO_2_max (r = 0.888), HR (r = 0.817), and lactate (r = 0.636, *p* < 0.01). There was no significant correlation with the duration of exercise (r = 0.342, *p* > 0.05) (Table 4).

## 4. Discussion

This study compared the effects of Kinesio taping on BF, exercise performance, and recovery in a high-temperature environment. The main finding is that, despite the observed increase in BF, KT had no significant effects on exercise performance or recovery.

### 4.1. Blood Flow Response

The results demonstrated a statistically significant increase in BF under the KTC condition compared to the UTC condition (*p* < 0.05). This aligns with previous studies suggesting that Kinesio taping’s mechanical properties, such as lifting the skin and reducing pressure on blood vessels, enhance local circulation [4]. Enhanced BF can facilitate better oxygen and nutrient delivery to active muscles during exercise.

However, the absence of significant interaction effects between time and condition indicates that this increase in BF was not consistent across all time points, resulting in no substantial differences in exercise intensity or recovery. Furthermore, contradictory findings exist, with some research suggesting that Kinesio taping does not significantly affect BF [17]. Therefore, additional research considering environmental conditions and participant characteristics is needed to clarify these effects.

During heat stress, the body prioritizes thermoregulation by increasing skin blood flow to facilitate heat dissipation [21]. Additionally, dehydration, which reduces plasma volume, can further impair cardiovascular and thermoregulatory responses [22]. This diminished plasma volume may exacerbate the reduction in muscle blood flow, thereby affecting exercise performance and the efficacy of kinesiology taping (KT). Therefore, in studies where hydration status is not controlled, interpreting the effects of KT on cardiovascular and thermoregulatory responses becomes challenging due to these confounding factors.

The BF response in high-temperature environments warrants further consideration. Under such conditions, blood redistribution prioritizes thermoregulation, which may limit the increase in muscle-blood flow even if KT enhances skin BF. Factors such as dehydration, electrolyte imbalance, and heat-induced fatigue may restrict overall exercise performance despite localized increases in BF due to taping [23]. While this study was conducted in a high-temperature environment, the exercise duration may not have been sufficient for heat stress to exert its full effect. Future studies should employ advanced imaging techniques, such as Doppler ultrasound, to explore vascular mechanisms and assess BF responses under varying conditions of high-temperature, exercise intensity, and duration.

### 4.2. Aerobic Performance and Heart Rate Response

Despite the observed increase in BF, no significant interaction effects between group and time were identified for VO_2_max or HR in the KTC and UTC conditions. This finding suggests that KT does not influence aerobic performance in high-temperature environments. While some studies have reported performance enhancements with KT, these effects are generally limited to muscular function improvement and do not extend to aerobic capacity [8,24].

Aerobic performance improvements, including peripheral circulation and VO_2_max, are primarily determined by factors such as muscle activation and systemic metabolic responses. However, during aerobic exercise, enhancements are more likely driven by central cardiovascular adaptations, such as increased stroke volume and cardiac output, rather than localized increases in BF attributed to taping [25]. Therefore, while KTC demonstrated the potential to improve localized circulation, it did not translate into enhanced aerobic performance under heat conditions. Future research should explore whether different taping methods, prolonged exercise durations, or varying environmental and physiological stress levels might influence aerobic capacity outcomes.

### 4.3. Blood Lactate and Recovery

In both conditions, BL levels increased immediately after exercise and decreased during recovery. However, the recovery rate of BL showed no significant differences between the KTC and UTC conditions. Lactate clearance is influenced by factors beyond localized muscle BF, including systemic circulation, oxidative metabolism, and the functions of the liver and kidneys [26]. This underscores the importance of systemic circulation in lactate clearance during recovery [27].

These findings suggest that while KT enhances peripheral circulation, it does not sufficiently impact systemic pathways involved in lactate metabolism. Interestingly, BL levels were higher in the KT condition during post-exercise, possibly indicating increased local muscle activation or metabolic demand. This finding may contradict the assumptions about KT facilitating recovery and suggests that the enhanced BF may not be sufficient to offset increased metabolic stress. In contrast to previous studies conducted under neutral conditions, our results provide context-specific insights into KT during heat stress, offering a more ecologically valid perspective on its practical application in real-world performance settings. As a result, the effects of KT on localized BF have a limited influence on overall lactate clearance and recovery.

### 4.4. Perceived Exertion and Heat Sensation

This study found no significant differences between the KTC and UTC conditions in perceived exertion or heat sensation. This suggests that, despite the observed physiological effects on BF, KT did not subjectively improve comfort or enhance exercise performance. Perceived exertion is influenced by a combination of physiological signals and cognitive factors [28]. Since participants reported no noticeable changes, the physiological benefits of KT may not have been translated into subjective perceptions. Similarly, trends in heat sensation and tympanic temperature showed no significant differences between the conditions, indicating that KT does not significantly affect thermoregulatory responses. This is likely because thermoregulation during exercise is primarily governed by systemic processes, such as skin BF and sweating [29], which are not significantly influenced by the mechanisms of KT.

### 4.5. Relationship Between Blood Flow and Physiological and Perceived Responses

In this study, correlations were observed between BF and variables such as aerobic capacity, HR, lactate levels, and perceived responses across all groups. BF during exercise is known to influence aerobic performance, HR, and perceived exertion [30,31,32]. However, since no significant differences were found between the KTC and UTC, these effects cannot be specifically attributed to the application of KT. Further studies conducted under more controlled and targeted research conditions are needed to explore these relationships in greater depth.

## 5. Limitations

The findings confirm that KT improves peripheral BF during exercise in hot conditions; however, its effects on aerobic performance, recovery, and subjective responses appear to be limited. Athletes and practitioners may consider KT as a tool to enhance localized circulation, particularly in rehabilitation or recovery settings. However, its effectiveness in improving performance or efficiency during aerobic exercise remains unproven. In high-temperature environments, additional research is required to account for variables such as thermoregulation, systemic metabolism, and fatigue recovery. While taping may serve as a supportive measure for exercise performance, its utility as a primary method for fatigue recovery or performance enhancement may be restricted. The study suggests that strategies to optimize thermoregulation and prevent dehydration are necessary to maximize the potential effects of taping under high heat stress. Further research is needed to identify the optimal conditions for KT to exert its effects.

It is worth noting that this study builds upon previous literature by examining the effects of KT under high-temperature exercise conditions, which has received limited attention. By addressing thermoregulatory and metabolic responses, it aims to provide new insights into the applicability of KT in heat-stress environments. Unlike earlier studies conducted under neutral conditions, our findings offer more practical and real-world relevant insights into the performance-related effects of KT during heat stress.

This study has several limitations that should be considered when interpreting the findings. The small sample size limited the statistical power and generalizability of the results. The sample size was constrained by limited access to participants meeting the inclusion criteria and strict environmental control requirements, which made large-scale recruitment challenging. Future studies with larger and more diverse populations are warranted to validate these findings. Additionally, all participants were healthy, physically active males, which may not reflect outcomes in other populations, such as female athletes or individuals with clinical conditions. Gender differences in thermoregulation and hormonal responses may yield different outcomes during exercise are well-documented, with variations in sweating rates, core temperature regulation, and hormonal fluctuations between males and females. For instance, females typically exhibit lower sweat output compared to males, which can influence heat dissipation during physical activity. Additionally, hormonal variations, such as those occurring during different phases of the menstrual cycle, can affect thermoregulatory mechanisms and metabolic responses to exercise. These physiological differences may lead to varied outcomes in exercise performance and adaptation between genders [33,34,35]. Furthermore, the controlled laboratory conditions, including temperature and humidity, may not fully replicate real-world exercise or training environments. Future research should address these limitations by recruiting larger and more diverse populations, including female participants and individuals with varying levels of fitness or health conditions. Studies should also investigate a broader range of environmental conditions and different types of exercise, employ more precise methods for monitoring core temperature, and ensure proper hydration control to improve the accuracy of findings. Additionally, further investigation into the molecular and hemodynamic mechanisms underlying KT is an important area for future research. Lastly, the lack of blinding is a notable limitation, as it may have influenced performance outcomes through expectancy or placebo effects.

## 6. Conclusions

In high-temperature conditions, KT significantly improved blood flow during and after exercise, indicating its potential benefits for enhancing localized circulation. However, KT did not influence aerobic performance, lactate clearance, or perceived exertion, suggesting limited systemic physiological impact during incremental aerobic exercise. These results imply that while KT may serve as a supportive strategy for promoting peripheral blood flow, it should not be relied upon as a primary method for improving aerobic performance. Practitioners and athletes may consider using KT as a supplementary tool for circulation improvement, but it is insufficient as a standalone method for enhancing performance in aerobic exercise.

## Figures and Tables

**Table 1:** Participant characteristics.

Age (yr)	Height (cm)	Weight (kg)	SMM (kg)	BF (%)	BMI (kg/m^2^)
25.7 ± 2.1	173.2 ± 5.8	76.7 ± 7.9	35.3 ± 3.6	19.3 ± 5.0	25.5 ± 2.2

SMM—skeletal muscle mass; BF—body fat content; BMI—body mass index; Values are mean ± SD.

**Table 2:** Measured environmental conditions.

Conditions		Start			End		
KTC	Temp (℃)	35.4	±	0.6	35.0	±	0.7
	Hum (%)	50.0	±	3.0	52.3	±	3.0
UTC	Temp (℃)	35.2	±	1.0	35.4	±	0.5
	Hum (%)	51.7	±	1.7	53.4	±	3.7

Temp—temperature; Hum—humidity; KTC—Kinesio taping condition; UTC—untreated condition. Values are mean ± SD.

**Table 3:** Comparison of rating of perceived exertion, thermal sensation, and eardrum temperature between KTC and UTC.

Parameter	Phase	KTC	UTC	Post Hoc
Rating of Perceived Exertion (6~20 Scale)	Baseline ^a^	7.9 ± 1.8	7.2 ± 1.4	a < b, c, d, e, f *** b < d, e *** c < d, e *** d > e, f *** e > f ***
	Rest ^b^	10.2 ± 1.8	10.1 ± 2.0	
	Ex ^c^	10.2 ± 1.5	10.3 ± 2.1	
	Post_Ex ^d^	17.7 ± 1.6	17.2 ± 1.3	
	Rec1 ^e^	13.1 ± 1.2	13.4 ± 2.1	
	Rec3 ^f^	11.0 ± 1.5	11.0 ± 1.6	
Thermal Sensation (−3~+3 Scale)	Baseline ^a^	−0.5 ± 0.7	−0.7 ± 0.8	a < b, c, d, e, f *** b < d ***, e *, b > f * c < d ***, c > f * d > e, f *** e > f ***
	Rest ^b^	1.4 ± 0.8	1.5 ± 0.7	
	Ex ^c^	1.3 ± 0.7	1.9 ± 0.6	
	Post_Ex ^d^	2.9 ± 0.7	2.8 ± 0.4	
	Rec1 ^e^	1.8 ± 0.3	2.2 ± 1.0	
	Rec3 ^f^	0.9 ± 0.9	1.0 ± 0.9	
Eardrum Temperature (℃)	Baseline ^a^	36.8 ± 0.4	36.7 ± 0.5	a < c *, d, e, f *** b < d, e ***, f ** c < d, e ***, f ** d < e * e > f ***
	Rest ^b^	36.9 ± 0.3	36.9 ± 0.3	
	Ex ^c^	37.0 ± 0.3	37.0 ± 0.3	
	Post_Ex ^d^	37.4 ± 0.4	37.4 ± 0.4	
	Rec1 ^e^	37.7 ± 0.3	37.7 ± 0.3	
	Rec3 ^f^	37.3 ± 0.2	37.3 ± 0.3	

Ex—exercise; Rec1—recovery phase 1; Rec3—recovery phase 3; KTC—Kinesio tape condition; UTC—untreated condition. a—baseline; b—rest; c—exercise; d—post-exercise; e—recovery phase 1; f—recovery phase 3. Values are mean ± SD. Significant difference is denoted as * *p* < 0.05; ** *p* < 0.01; *** *p* < 0.001.

**Table 4:** Correlations between blood flow, physiological and subjective indicators, and exercise duration.

Parameter	Blood Flow	
	KTC	UTC
VO_2_max	0.883 **	0.888 **
Heart Rate	0.774 **	0.817 **
Rating of Perceived Exertion	0.651 **	0.672 **
Thermal Sensation	0.581 **	0.592 **
Eardrum Temperature	0.285 *	0.367 *
Lactate	0.749 **	0.636 **
Duration	−0.1	0.342

KTC—Kinesio tape condition; UTC—untreated condition. Significant difference is denoted by: * *p* < 0.05, ** *p* < 0.01.

## Data Availability

The data presented in this study are available on request from the corresponding author. The data are not publicly available due to the protection of personal information.
